# Increased NHC Cells in the Peritoneal Cavity of Plasmacytoma Susceptible BALB/c Mouse

**DOI:** 10.1155/2015/313140

**Published:** 2015-10-04

**Authors:** Berenice Sánchez-González, Francisco Javier García-Vázquez, José Eduardo Farfán-Morales, Luis Antonio Jiménez-Zamudio

**Affiliations:** ^1^Departamento de Inmunología, Laboratorio de Inmunología Clínica, Escuela Nacional de Ciencias Biológicas, Instituto Politécnico Nacional, 11340 Mexico, DF, Mexico; ^2^Departamento de Anatomía Patológica, Laboratorio de Patología Molecular, Instituto Nacional de Pediatría, 04530 Mexico, DF, Mexico

## Abstract

BALB/c strain mice are unique in that they develop murine plasmacytoma (MPC) as a consequence of the inflammation induced by pristane oil injection in the peritoneal cavity. In this work the Treg, Th17, B1, B2, and NHC lymphocyte populations from the peritoneal environment of BALB/c, the susceptible strain, and C57BL/6 mice, which do not develop MPC after oil treatment, were studied. Both oil-treated strains showed decreased levels of Th17 lymphocytes, no significant variation in Treg lymphocytes, and a drastic decrease of all B lymphocyte populations. However, only oil-induced BALB/c showed increased levels of natural helper cells (NHC) which could be important in the myeloma induction.

## 1. Introduction

BALB/c mice develop murine plasmacytoma (MPC) upon intraperitoneal administration of pristane, (2,6,10,14-tetramethylpentadecane), a component of mineral oil [1–5]. IL-6, TGF-*β*1, and other factors are required for transformation of B lymphocyte to MPC [6–8]. IL-6 blocks the differentiation of the Treg cells [9, 10] which inhibit autoreactive T cell and modulate immune functions by releasing TGF-*β*1 and IL-10 [11], but it promotes the generation of proinflammatory Th17 cells, characterized by the expression of ROR *γ*t transcription factor and the production of IL-17, which induces the production of a wide range of proinflammatory cytokines and neutrophil-attracting chemokines [12].

The innate lymphoid cell population which has been called natural helper cells (NHC) for its cytokine profile may also be involved in the inflammatory process [13]. These cells are found in lymphoid clusters surrounded by fatty tissue, which have been called FALCs (of fat-associated lymphoid cluster), and have markers such as c-kit, Sca-1, IL7R, and IL33R [13–17]. NHC are involved in the B1 cells proliferation [13].

The B1 lymphocytes are a B cell subpopulation, markedly different from the usually studied B2. Whereas B2 are CD23^+^ and CD43^−^, B1 cells are CD23^−^ and CD43^+^. B1 cells are further divided into B1a (CD5^+^) cells and B1b (CD5^−^). B1 are self-renewing and are found in the peritoneal and pleural cavities and its main function is the constitutive production of natural IgM antibodies, which provide early protection to a variety of pathogens [18–21]. B1a cells have regulatory properties because their IL-10 production [21–24] and promote expansion of T cells [25, 26]. However, B1 cells are often autoreactive [18], but it is not well understood how they are regulated to prevent autoimmunity.

The aim of the study was to determine what is it that makes the BALB/c susceptible to the myeloma development as a consequence of the inflammatory response induced by the pristane oil in the peritoneal cavity.

## 2. Methods

### 2.1. Mice

BALB/cAnNHsd and C57BL/6NHsd female mice, 7-8 weeks old, were purchased from Harlan Laboratories, S.A. de C.V. Mice were handled at all times according to the Official Mexican Standard NOM-062-Z00-1999, technical specifications for production, care and use of laboratory animals. Pristane injections did not require the use of anesthetic, analgesic, or tranquilizer. The intraperitoneal administration of pristane was performed in the animal while always handling it gently but firmly, avoiding the struggle and stress at all times. The method used for euthanasia of mice was exposure to carbon dioxide (CO_2_).

### 2.2. Oil Induction

Mice were injected intraperitoneally with 500 *μ*L pristane (P2870, Sigma-Aldrich), at days 0, 60, and 120. We used the method as described by Potter and Wax, of 1.5 mL pristane given in 3 0.5 mL doses spaced 2 months apart [5].

### 2.3. Obtention of Peritoneal Content and Mesenteric and Mesothelial Tissues

Seven months after the initial pristane treatment, the oil-treated and control mice of both mouse strains were sacrificed and the peritoneal content was obtained as previously described [27]. A piece of skin was cut into the belly to expose the mesothelium and the peritoneal cavity was washed by introducing 5 mL of RPMI 1640 medium (31800-022 Gibco). The cavity was vigorously massaged and the content was aspirated using the same syringe. The oil-treated BALB/c mice develop MPC after seven months and in this group the peritoneal content was aspirated directly due to large accumulation of ascitic fluid. From the peritoneal content cells and fluid were separated by centrifugation. Obtention of mesenteric tissue and identification of FALCs regions (fat-associated lymphoid clusters of NHC) with toluidine blue were performed as described [13]. Nevertheless, in oil-treated mice of both strains the FALCs regions were not visibly detectable. Mesenteric and mesothelial tissues were collected in both strains only at seven months after induction and preserved in paraffin blocks [1, 28, 29].

### 2.4. Lymphocyte Determination by Flow Cytometry

Cells were determined from the peritoneal content obtained from control and oil-induced mice at seven months. For Th17 determination cells were stimulated during four hours in the presence of 50 ng/mL of PMA (Phorbol 12-myristate 13-acetate P 8139, Sigma) and 500 ng/mL of Ionomycin (Ionomycin calcium salt 10634, Sigma-Aldrich). For the last two hours, Brefeldin A (00-4506, eBioscience) and Protein Transport Inhibitor (554724, BD GolgiStop) were added. Treg and Th17 cells were preincubated with Purified Rat Anti Mouse CD16/CD32 clone 2.4G2 (553142, BD Pharmingen) to block Fc receptors. Subsequently the extracellular staining was performed in 100 *μ*L of PBS-F (PBS/2% FACS Buffer) and the specific antibodies, for 30 minutes in darkness. For Treg and Th17 cell identification, PerCP Hamster Anti-Mouse CD3ɛ clone 145-2C11 (553067, BD Pharmingen) and FITC Rat Anti-Mouse CD4 clone RM4-5 (553046, BD Pharmingen) and, for B1 cells, PerCP Rat Anti-Mouse CD5 clone 53-7.3 (553025, BD Pharmingen), FITC Rat Anti-Mouse CD43 clone S7 (553270, BD Pharmingen), PE Rat Anti-Mouse CD11b clone M1/70 (553311, BD Pharmingen), and APC Rat Anti-Mouse CD19 clone 1D3 (550992, BD Pharmingen) were used. Prior to intracellular staining, Treg and Th17 cells were fixed for 30 minutes at 4°C and permeabilized for 30 minutes at 37°C both in the dark with Mouse Foxp3 Buffer Set (560409, BD Pharmingen). Intracellular staining of Th17 and Treg cells was performed with the PE Rat Anti-Mouse IL-17A clone TC11-18H10 (559502, BD Pharmingen) and PE Rat Anti-Mouse Foxp3 clone MF23 (560408, BD Pharmingen), respectively, for 30 minutes in a volume of 100 *μ*L PBS-F in the dark. Finally, cells were analyzed on the FACSAria cytometer, using the Davia software.

### 2.5. NHC Cells and Malignant Plasma Cells

Nine-micron sections were cut from the mesenteric and mesothelial tissue preserved in paraffin blocks. Histological sections were subjected to a hydration and unmasking treatment, and specific staining with the corresponding primary and secondary antibody was performed. The primary antibody for the identification of NHC cells was CD117/c-kit clone EP10 (CME 296 AK, Biocare Medical) and Biotin-SP-AffiniPure Goat Anti-Rabbit IgG (111-065-003, Jackson ImmunoResearch) as the secondary antibody. Malignant plasma cells were stained with Purified Rat Anti-Mouse CD138 clone 281-2 (553712, BD Pharmingen) and Peroxidase-AffiniPure Goat Anti-Rat IgG (112-035-062, Jackson ImmunoResearch).

### 2.6. Cytokines Assay

Cytokines were determined from the peritoneal fluid from both mouse strains, control and oil-induced at seven months, with commercial kits of eBioscience: Mouse IL-6 ELISA Ready- Set-Go (88-7064), Human/Mouse TGF beta 1 ELISA Ready-SET-Go! 2nd Generation (88-8350), Mouse IL-10 ELISA Ready-SET-Go! 2nd Generation (88-7105), Mouse IL-4 ELISA Ready-SET-Go! (88-7044), Mouse IL-9 ELISA Ready-SET-Go! 2nd Generation (88-8092), Mouse IL-13 ELISA Ready-SET-Go! (88-7137), Mouse IL-5 ELISA Ready-SET-Go! (88-7054), Mouse IFN gamma ELISA Ready-SET-Go! (88-7314), and Mouse IL-33 ELISA Ready-SET-Go! (88-7333).

### 2.7. Statistical Analysis

The results of each test are represented by the median of five or more independent experiments. The nonparametric Mann-Whitney* U* was utilized by GradPad Prisma program for displaying graphics and Sigma Plot 12.0 for analysis considering significant differences in value *P* ^*∗*^ ≤ 0.05, *P* ^*∗∗*^ ≤ 0.008, and *P* ^*∗∗*^ ≤ 0.005

## 3. Results

### 3.1. MPC in BALB/c Mice

**Figure 1:**
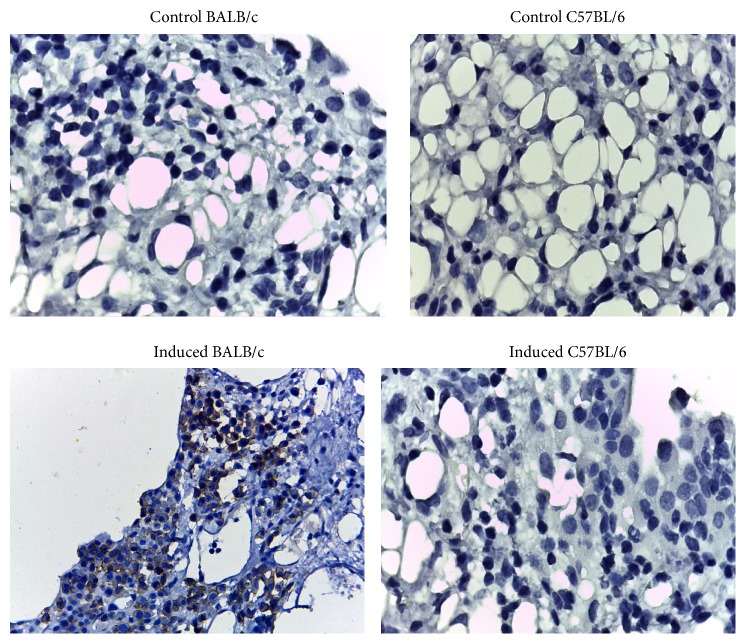
Mesothelial tissue of control and oil-induced mice from both mouse strains at seven months after induction. Only the pristane-induced BALB/c mouse developed malignant plasma cells CD138^+^ (cells with brown tonalities membrane), 40x magnification.

The tumor cells were present in the mesothelium and in nodular whitish tissue from BALB/c oil-treated mice and were identified by the presence of CD138^+^ (Figure 1).

### 3.2. Th17 and Treg Cells Determination

**Figure 2:**
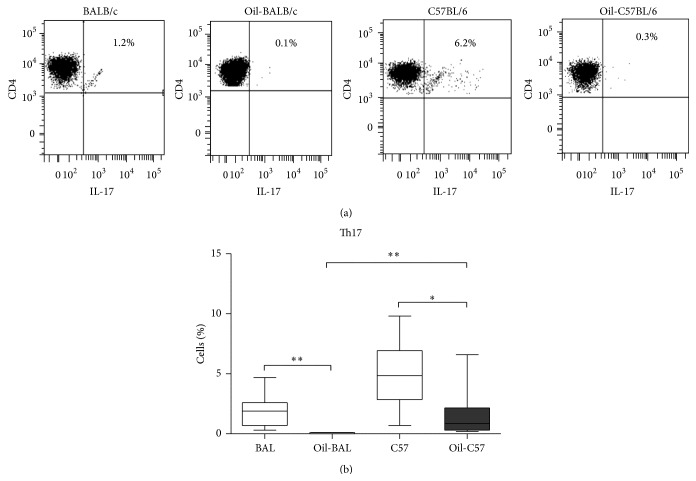
Th17 cells. (a) Dot plots show percentages of Th17 cells from the peritoneal content from control and oil-induced mice at seven months after induction. The CD4^+^IL-17^+^ cells come from CD3^+^ selected from total lymphocyte region. (b) Percentage medians of Th17 without activation in the aforementioned groups of animals *P* ^*∗*^ ≤ 0.05, *P* ^*∗∗*^ ≤ 0.008 (*n* = 5–10). Th17 cells of induced animals (oil-induced) of both strains are decreased, but the percentage is higher in the oil-induced C57BL/6 strain compared with oil-induced BALB/c strain.

**Figure 3:**
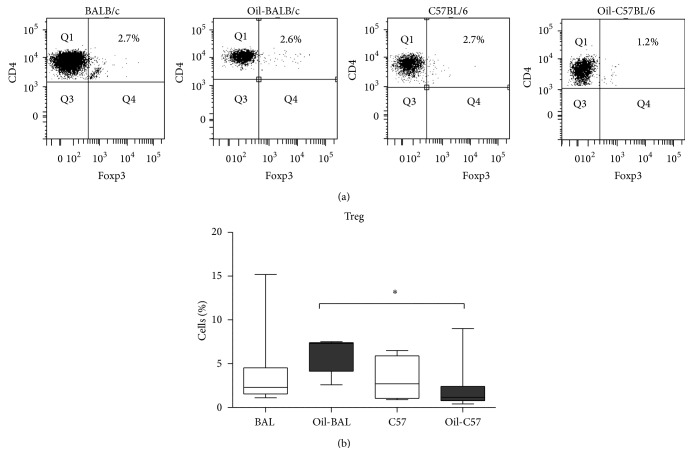
Treg cells. (a) Dot plots show percentages of Treg cells from the peritoneal content from control and induced mice at seven months after induction. The lymphocytes CD4^+^Foxp3^+^ derived from CD3^+^ selected from total lymphocyte region. (b) Percentage medians of Treg in the above groups of animals *P* ^*∗*^ ≤ 0.05, *P* ^*∗∗*^ ≤ 0.008 (*n* = 5–10). No changes were observed in Treg when animals were induced. However, the BALB oil-induced mice had a higher percentage of Treg than the C57BL/6 oil-induced mice.

Treg (Foxp3^+^CD3^+^CD4^+^) and Th17 (CD3^+^CD4^+^IL-17^+^) cells were determined by flow cytometry in both strains, seven months after oil induction and in control mice. For Th17 determination the cells were activated with PMA/Ionomycin. Not activated Th17 cells were reduced in both strains of induced mice (Figures 2(a) and 2(b)), although a higher percentage of Th17 cells was found inthe oil-induced C57BL/6 mice in comparison with oil-induced BALB/c (Figure 2(b)). The differences in Treg cells analyzed at seven months after induction were not significant. However when the induced mice strains were compared, the percentage of Treg cells was higher in BALB/c (Figure 3(b)).

### 3.3. B1 Cells Determination

**Figure 4:**
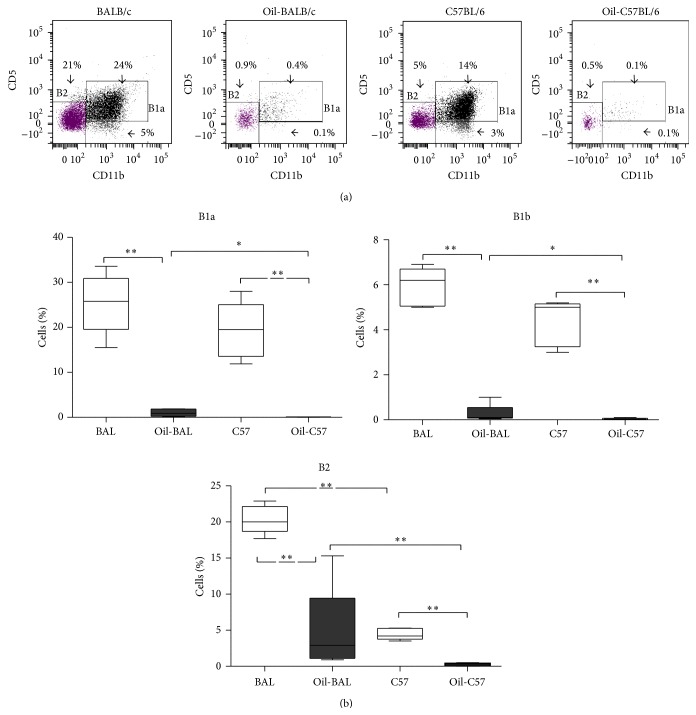
B2, B1a, and B1b cells. (a) Dot plots show percentages of B cells from the peritoneal content from control and induced mice at seven months after induction of BALB/c and C57BL/6 strains. CD5^−^ CD11b^−^ (B2 lymphocytes), CD5^+^ CD11b^+^ (B1a lymphocytes), and CD5^−^ CD11b^+^ (B1b lymphocytes) derived from CD19^+^ selected from total lymphocyte region. (b) Percentage medians of B lymphocytes in the above groups of animals *P* ^*∗*^ ≤ 0.05, *P* ^*∗∗*^ ≤ 0.008 (*n* = 5–10). All subpopulations of B lymphocytes of oil-induced animals of both strains are diminished. BALB/c oil-induced mice present higher B1a, B1b, and B2 with respect to the C57BL/6 oil-induced mice. BALB/c control mice show a greater percentage of B2 with respect to the C57BL/6 control mice.

The B1a (CD19^+^43^+^CD11b^+^CD5^+^), B1b (CD19^+^43^+^CD11b^+^CD5^−^) [19, 30, 31], and B2 (CD19^+^) cells were determined from the peritoneal contents of both BALB/c and C57BL/6 oil-induced and control mice by flow cytometry at seven months after induction. All B cell populations were diminished in the oil-induced mice in both strains (Figures 4(a) and 4(b)).

### 3.4. NHC Cells Determination

**Figure 5:**
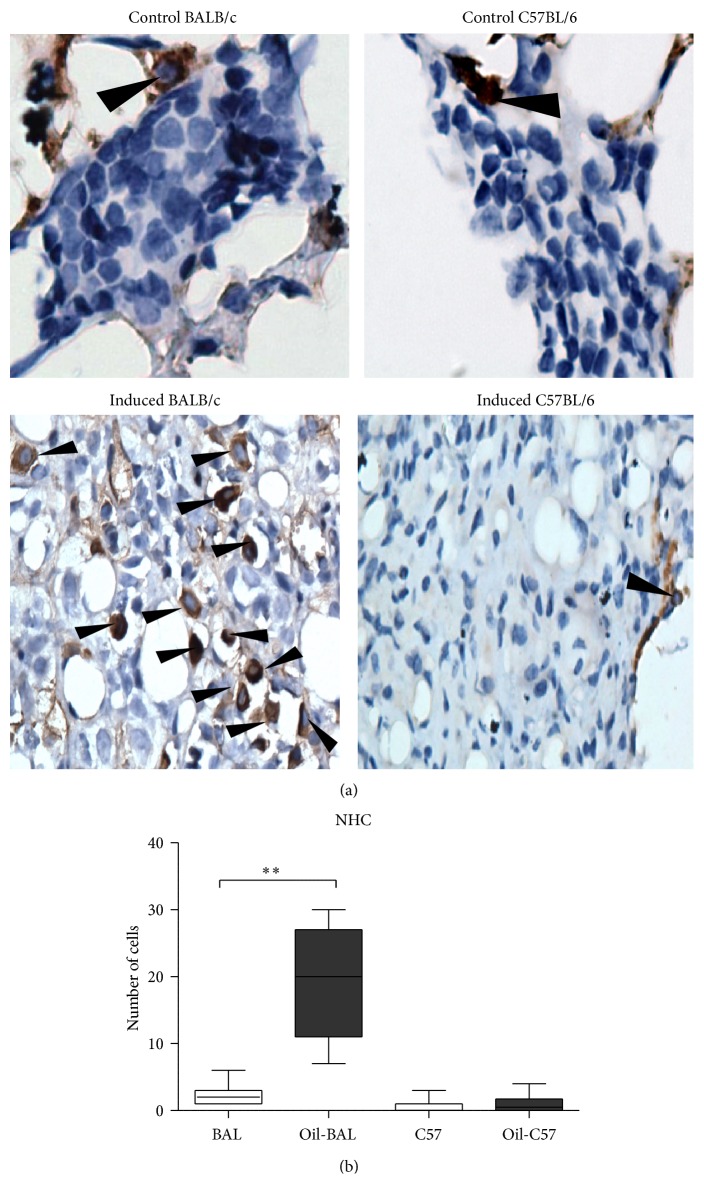
NHC cells. (a) Mesenteric tissue from control and oil-induced mice from both mouse strains at seven months after induction which show c-kit^+^ NHC cells (cells with brown tonalities membrane; see arrows), 40x magnification. (b) Medians of number NHC cells *P* ^*∗∗*^ ≤ 0.005 (*n* = 10). Increase of NHC cells was observed only in oil-induced BALB/c mice.

The NHC (c-kit^+^) cells were determined in the mesenteries of BALB/c and C57BL/6 mice at seven months after oil-induction. NHC cells were increased only in oil-induced BALB/c mice (Figures 5(a) and 5(b)).

### 3.5. Cytokines Determination

IL4, IL-5, IL-9, IL-13, INF-*γ*, IL-6, IL-10, IL-33, and TGF-*β* were determined in the peritoneal fluid from both strains at seven months after induction.  Table 1 shows no production at all of IL-4, IL-13, and IL-33, whereas IL-9 was equally produced by all groups. INF-*γ*, IL-6, and TGF-*β* levels (in green) are increased in oil-induced animals; however, no significant difference was found between BALB/c and C57BL/6 strains.

**Figure 6:**
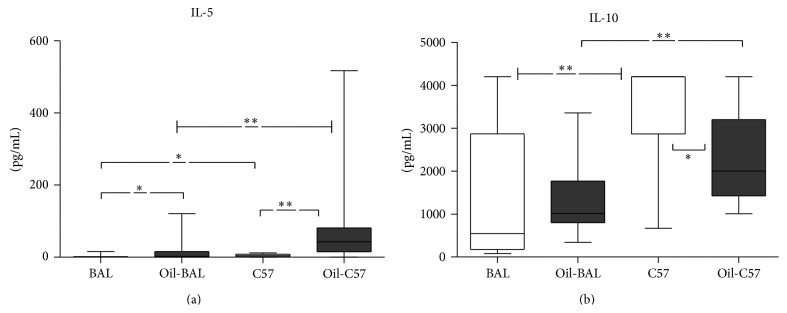
IL-5 and IL-10 cytokines. (a) Concentration medians of IL-5. (b) Concentration medians of IL-10. *P* ^*∗*^ ≤ 0.05, *P* ^*∗∗*^ ≤ 0.005 (*n* = 10–14).

Only in IL-5 and IL-10 a significant difference between strains was found (Figures 6(a) and 6(b) and Table 1, in pink). An increase of IL-5 was found in oil-induced mice, but the highest concentrations were found in C57BL/6. IL-10 was higher in both control and induced C57BL/6 mice as compared with BALB/c mice, despite a decrease of this cytokine in oil-induced C57BL/6 mice, when compared with controls.

## 4. Discussion

It is well known that BALB/c is the only mouse strain susceptible to develop myeloma as result of an inflammatory reaction induced by the intraperitoneal injection of an irritant, usually pristane [4, 29].

To try to find out which is the unique characteristic of BALB that allows for the myeloma appearance, we injected pristane in mice of the susceptible BALB/c strain and mice of the not susceptible C57BL/6 strain as control, and determinations of several cells and cytokines were made at seven months after the oil treatment as well as in noninduced control animals.

Both BALB/c and C57BL/6 strains developed a chronic inflammatory process after the pristane treatment. Large lymphocyte infiltrates were observed in the peritoneal content of both induced strains but to a greater degree in BALB/c strain (data not shown).

The appearance of myeloma in BALB/c mice was observed seven months after pristane treatment, by the presence of malignant plasma cells with neoplastic morphology and the CD138^+^ marker as has been reported [28].

In this work it was found that the regulation of Th17 and Treg is different between oil-induced BALB/c mice and oil-induced C57BL/6 mice.

Th17 cells of both strains were reduced in oil-induced mice compared to controls, but there were no significant differences between Treg from pristane treated mice and controls, which indicates that the decrease of Th17 is not due to Treg. However when comparing both strains of oil-induced mice, a greater percentage of Treg cells and lower percentage of TH17 cells were observed in the oil-induced BALB/c mice, compared to oil-induced C57BL/6 mice which showed inversely proportional values. This would suggest a greater effort to try to control the inflammatory process in the BALB/c mouse.

We also found a decrease in subpopulations B1, both B1a and B1b, and B2 lymphocytes in both strains of oil-induced animals, which could be explained by the migration of these cells into other lymphoid compartments. Upon appropriate stimulus, B1 cells in the peritoneal cavity migrate to the mesenteric lymph nodes (MLNs), where they differentiate into antibody-secreting plasma cells [32, 33].

Furthermore, the effect of some growth factors generated in the same inflammatory niche and genes of BALB/c mouse as c-myc could influence the evolution of these B lymphocytes to malignant plasma cells [6].

An important difference between the strains was the increase of NHC cells only in BALB/c. Little is known about these cells, but its importance in the innate response has been reported and has been identified as an immunity promoting factor; however recent studies show that they are also involved in tumor formation in immune microenvironments. Bie et al. propose that these cells in peripheral blood are very closely related to an immunosuppressive microenvironment in gastric cancer [34]. This increase in NHC could be an innate response of BALB/c mouse for inflammation control; however, the high amount of these cells could influence the development of the myeloma.

Of the studied cytokines, only IL-5 and IL-10 exhibited significant differences between the two strains. The cytokines IL-4, IL-13, IL-33, IL-9, IFN-*γ*, IL-6, and TGF-*β* had irrelevant results (Table 1). The cytokines IL-4, IL-13, and IL-33 were not present or their levels were below our detection limits. IL-9 was detected, but no significant differences were found between oil-induced and control animals. Although there are reports of increased levels in pulmonary inflammation [35], IL-9 does not seem of relevance in the peritoneal inflammation.

INF-*γ*, IL-6, and TGF-*β* were increased as expected in the inflammation process in the induced animals; however, no differences were found between the strains.

IL-5 level was higher in oil-induced mice of both strains. As the receptor for IL-5 (IL-5R) shares the *β* chain with the GM-CSFR receptor, it can promote lymphopoiesis [36–38] and probably the increase of NHC. However, the increase in IL-5 was higher in C57BL/6 strain which had also higher levels in the control mice.

C57BL/6 showed a high normal level of IL-10 [39] and even after a decrease in the induced C57BL/6 the levels of IL-10 are still much higher than in the induced BALB/c.

Therefore, the high concentration of IL-10 found in the C57BL/6 mice could prevent the IL-5 induced increase in NHC, thus avoiding the MPC, in contrast with BALB/c in which the expansion of NHC lymphocytes could probably drive its development.

Multiple myeloma (MM) is a cancer of plasma cells in humans, and although there have been great strides in recent years in the study of this disease, MM cause remains unknown. Our work in the murine model suggests the involvement of the NHC as an important subject for further research but also as a potential target for an effective therapy.

## 5. Conclusions

The only important difference between the oil-induced inflammatory processes in the two strains is that whereas in the C57BL/6 mice there is no variation in NHC, these cells are increased in the BALB/c strain, which may be related with the surge of the myeloma.

## Figures and Tables

**Table 1:** Cytokines of peritoneal fluid from mice of both strains BALB/c and C57BL/6, control and induced with pristane, at seven months after induction.

Cytokines	Medians of concentration (pg/mL)			
	BALB/c		C57BL/6	
	Control	Oil-induced	Control	Oil-induced
IL-4	1	0	0	0
IL-13	0	0	0	0
IL-33	0	0	0	0
IL-9	78	72	73	71
*INF-γ*	*0 *	* 51* ^**^	*0 *	*59* ^**^
*IL-6 *	*0 *	*525* ^**^	*0 *	*314* ^**^
*TGF-β*	*0 *	* 1469* ^*^	*0 *	* 1408* ^*^
**IL-5**	**0**	** 2** ^*^	** 4**	** 43** ^**^
**IL-10**	** 544**	** 1015**	**4198** ^*^	** 2004 **

^*^ *P* ≤ 0.05, ^*∗∗*^ *P* ≤ 0.005 (*n* = 10–14).
